# Rates and Correlates of HIV Incidence in Namibia’s Zambezi Region From 2014 to 2016: Sentinel, Community-Based Cohort Study

**DOI:** 10.2196/17107

**Published:** 2020-06-24

**Authors:** Andrew D Maher, Tuli Nakanyala, Nicholus Mutenda, Karen M Banda, Dimitri Prybylski, Adam Wolkon, Anna Jonas, Souleymane Sawadogo, Charity Ntema, Melody Regina Chipadze, Grace Sinvula, Annastasia Tizora, Asen Mwandemele, Shaan Chaturvedi, Afiba Manza-A Agovi, Simon Agolory, Ndapewa Hamunime, David W Lowrance, Willi Mcfarland, Sadhna V Patel

**Affiliations:** 1 Institute for Global Health Sciences University of California San Francisco San Francisco, CA United States; 2 South African Centre for Epidemiological Modelling and Analysis Stellenbosch University Stellenbosch South Africa; 3 Directorate for Special Programs Ministry of Health and Social Services Windhoek Namibia; 4 Division of Global HIV and TB US Centers for Disease Control and Prevention Atlanta, GA United States; 5 Total Control of the Epidemic Development Aid from People to People Windhoek Namibia

**Keywords:** HIV, incidence, risk factors, sentinel surveillance, longitudinal studies, cohort studies

## Abstract

**Background:**

Direct measures of HIV incidence are needed to assess the population-level impact of prevention programs but are scarcely available in the subnational epidemic hotspots of sub-Saharan Africa. We created a sentinel HIV incidence cohort within a community-based program that provided home-based HIV testing to all residents of Namibia’s Zambezi region, where approximately 24% of the adult population was estimated to be living with HIV.

**Objective:**

The aim of this study was to estimate HIV incidence, detect correlates of HIV acquisition, and assess the feasibility of the sentinel, community-based approach to HIV incidence surveillance in a subnational epidemic hotspot.

**Methods:**

Following the program’s initial home-based testing (December 2014-July 2015), we purposefully selected 10 clusters of 60 to 70 households each and invited residents who were HIV negative and aged ≥15 years to participate in the cohort. Consenting participants completed behavioral interviews and a second HIV test approximately 1 year later (March-September 2016). We used Poisson models to calculate HIV incidence rates between baseline and follow-up and multivariable Cox proportional hazard models to assess the correlates of seroconversion.

**Results:**

Among 1742 HIV-negative participants, 1624 (93.23%) completed follow-up. We observed 26 seroconversions in 1954 person-years (PY) of follow-up, equating to an overall incidence rate of 1.33 per 100 PY (95% CI 0.91-1.95). Among women, the incidence was 1.55 per 100 PY (95% CI 1.12-2.17) and significantly higher among those aged 15 to 24 years and residing in rural areas (adjusted hazard ratio [aHR] 4.26, 95% CI 1.39-13.13; *P*=.01), residing in the Ngweze suburb of Katima Mulilo city (aHR 2.34, 95% CI 1.25-4.40; *P*=.01), who had no prior HIV testing in the year before cohort enrollment (aHR 3.38, 95% CI 1.04-10.95; *P*=.05), and who had engaged in transactional sex (aHR 17.64, 95% CI 2.88-108.14; *P*=.02). Among men, HIV incidence was 1.05 per 100 PY (95% CI 0.54-2.31) and significantly higher among those aged 40 to 44 years (aHR 13.04, 95% CI 5.98-28.41; *P*<.001) and had sought HIV testing outside the study between baseline and follow-up (aHR 8.28, 95% CI 1.39-49.38; *P*=.02). No seroconversions occurred among persons with HIV-positive partners on antiretroviral treatment.

**Conclusions:**

Nearly three decades into Namibia’s generalized HIV epidemic, these are the first estimates of HIV incidence for its highest prevalence region. By creating a sentinel incidence cohort from the infrastructure of an existing community-based testing program, we were able to characterize current transmission patterns, corroborate known risk factors for HIV acquisition, and provide insight into the efficacy of prevention interventions in a subnational epidemic hotspot. This study demonstrates an efficient and scalable framework for longitudinal HIV incidence surveillance that can be implemented in diverse sentinel sites and populations.

## Introduction

### Background

Namibia has a generalized epidemic with 237,000 adults (13.3%) living with HIV [1]. The prevalence varies by geography, ranging from 7.3% in the Omaheke region to 23.7% in the Zambezi region [2]. Namibia’s epidemic response is robust. The number of people living with HIV (PLHIV) on antiretroviral treatment (ART) increased from 10,200 in 2004 [3] to 166,000 in 2016 [4]. By 2022, Namibia seeks to reduce new HIV infections by 75% through scaling-up evidence-based interventions such as medical male circumcision, viral suppression through ART for all PLHIV, and pre-exposure prophylaxis (PrEP) in high-burden regions [5].

Namibia, like most countries with generalized epidemics, has limited ability to assess the impact of prevention interventions and monitor HIV incidence over time. The gold standard for measuring HIV incidence is a longitudinal cohort study, which entails enrolling persons uninfected at baseline and following them over time with repeated testing to detect acquisition of infection. Owing to the perceived high cost and logistical complexity, few surveillance cohort studies have been conducted around the world in recent years [6-10]. Alternative approaches to estimate incidence, including mathematical models [11-13] and assays for recent infection [14], are available. However, models depend on assumptions that are difficult to prove, do not establish causality, and are imprecise at subnational levels. Assays for recent infections have multiple sources of variability, which necessitate large sample sizes and correction factors [14].

A pragmatic method for tracking HIV incidence may be found in the sentinel approach to surveillance [15,16], which involves using data from selected clinics, facilities, or programs. The program’s clientele, while not necessarily representative of everyone at risk, is held to reflect changes in the epidemic in the surrounding population. Community-based HIV testing programs, now common in many regions of sub-Saharan Africa, may provide a platform for sentinel incidence surveillance [17,18]. Home, mobile, workplace, and school-based programs can increase testing in populations, including repeat testing, by removing social and logistical barriers associated with testing at facilities [19-21]. Therefore, the basic infrastructure for longitudinal sentinel incidence surveillance may already be present in certain high-prevalence areas.

### Objectives

We conducted a sentinel HIV incidence cohort study by adding behavioral measurements and repeated testing to an existing community-based program offering home testing in Namibia’s Zambezi region. Our objectives were to estimate HIV incidence, detect new or confirm known risk and preventive factors for HIV acquisition, and assess the feasibility of the sentinel approach to HIV incidence surveillance in a subnational epidemic hotspot.

## Methods

### Study Setting and Design

The study was a prospective cohort implemented in households in Namibia’s Zambezi region, situated in the northeast bordering Angola, Botswana, Zambia, and Zimbabwe. Zambezi was chosen because it has the highest prevalence of HIV in the country (23.7%) [2]. Additionally, a community-based program, Total Control of the Epidemic (TCE), initiated HIV testing and case management for residents of all households in the Zambezi region (20,603 people, 2011 Census) in 2014. TCE’s home-based program entailed HIV testing and prevention plans focusing on abstinence, being faithful to 1 partner, condom use, medical male circumcision, repeated testing every 6 to 12 months, and referrals to ART with case management for HIV-positive clients.

TCE mapped all households in the Zambezi region and divided them into 60 programmatic *fields*, each composed of 6 to 7 geographically contiguous clusters of 60 to 70 households. We selected 1 cluster from each of the 10 fields to include in the sentinel incidence cohort. Clusters were purposively selected to include urban or rural areas of varying distance from the regional capital (Katima Mulilo). Adjacent clusters were paired to form 5 study *sites*. All households in the sites were eligible for the study. Cohort activities were integrated into the routine activities of TCE’s program as they worked on these sites. The cohort aimed to enroll and complete a 1-year follow-up of 1500 persons to obtain reasonably precise HIV incidence estimates and sufficient power to identify strong correlates of seroconversion.

### Recruitment and Procedures

TCE staff approached all households in the sites to offer home-based HIV testing to all residents from December 2015 to July 2016. Residents were identified by the head of the household and assigned unique testing codes. GPS coordinates were recorded at each household to facilitate household identification. Residents aged ≥15 years who received the TCE program were invited to complete a baseline interview. Clients who tested negative for HIV were invited to participate in the cohort.

Data on exposure to prevention interventions (eg, HIV testing outside the study, ART use in serodiscordant partnerships, and medical male circumcision), HIV-related risk and preventive behaviors (eg, multiple partners and transactional sex), and demographic characteristics (eg, sex, age, and marital status) were obtained in face-to-face interviews.

Rapid HIV testing was done in the participant’s household by TCE staff following the national parallel algorithm, including Alere Determine HIV-1/2 (Abbott Diagnostic Division) and Uni-Gold Recombigen HIV-1/2 (Trinity Biotech) with Clearview Complete HIV-1/2 (Inverness Medical) to resolve discrepant results. Results from the rapid testing algorithm were immediately returned to participants with posttest counseling.

TCE staff collected dried blood spot (DBS) specimens from participants by finger prick on Whatman 903 filter paper. DBS were dried and packaged according to the manufacturer’s instructions and shipped weekly to the National Institute of Pathology reference laboratory in Windhoek and stored at −70°C to −80°C. A fourth-generation enzyme-linked immunosorbent assay (Vironostika Uniform II bioMérieux-Diagnostics) was used on DBS for quality assurance to confirm every 10th HIV-negative and all HIV-positive rapid test results at baseline and follow-up. Quality assurance results were not returned to the participants. Additional quality assurance was performed according to national standards, including proficiency panels for counselors throughout the study.

Cohort participants were recontacted approximately 12 months after enrollment to complete a follow-up interview and HIV test using the same procedures.

### Statistical Analysis

Proportions and 95% CIs were calculated to describe the characteristics of the cohort. We used baseline interview data for demographic characteristics, prior testing history, partner’s HIV status, and male circumcision. We used follow-up interview data for variables that may have changed from baseline to follow-up, including seeking testing for HIV outside of the study, transactional sex, sex with partners residing outside of the study sites, condom use, and multiple sex partners. We used generalized linear models to assess baseline correlates of cohort participation and completion of follow-up.

Rates of HIV incidence were calculated as the number of seroconversions per 100 person-years (PY) of follow-up. PY was calculated as the number of days between baseline and follow-up/365 for participants who did not seroconvert and one-half the number of days between baseline and follow-up/365 for participants who seroconverted, which is a commonly used technique when the exact date of seroconversion is unknown [7,22,23]. To account for possible dependence among participants in the selected field sites, we used the *field* variable to calculate cluster-robust 95% CI for incidence rates [24], except when a variable’s strata contained 1 or no seroconversions. For these cases, the exact 1- or 2-sided Poisson CI was calculated. We used Cox models to assess potential correlates of HIV seroconversion. Since patterns of intergenerational heterosexual transmission resulting in a higher HIV incidence among young women and older men have been observed elsewhere in sub-Saharan Africa, along with different risk factors for HIV infection prevailing for men and women [1,8,9,25], we modeled data among men and women separately. Variables that had zero seroconversions or failed to meet the proportional hazard assumption were excluded. Variables that produced *P* values <.10 in the bivariate models were included in the initial multivariable models. We used the variance inflation factor with Stata’s *vif* command to assess the potential for multicollinearity of variables [26]. Any variable with a variance inflation factor greater than 10 was excluded from the multivariable model. Variables with *P*<.05 in the final models were considered significant. The risk of seroconversion was expressed as adjusted hazard ratios (aHR). The analysis was performed using Stata version 12.1 (StataCorp).

### Ethical Information

Participants gave verbal informed consent at baseline and again at follow-up. Participants aged 15 to 17 years gave their assent and were required to have consent from a parent or guardian. No monetary or material incentives were provided. The study was approved by the Institutional Review Boards of the Ministry of Health and Social Services in Namibia and the University of California, San Francisco. The study was reviewed in accordance with the Centers for Disease Control and Prevention (CDC) human research protection procedures and determined to be research, although CDC investigators did not interact with human subjects or have access to identifiable data or specimens for research purposes. All procedures were implemented in accordance with the ethical standards of the abovementioned ethics committees and the Helsinki Declaration of 1975, as revised in 2000.

## Results

### Participation and Retention

**Figure 1:**
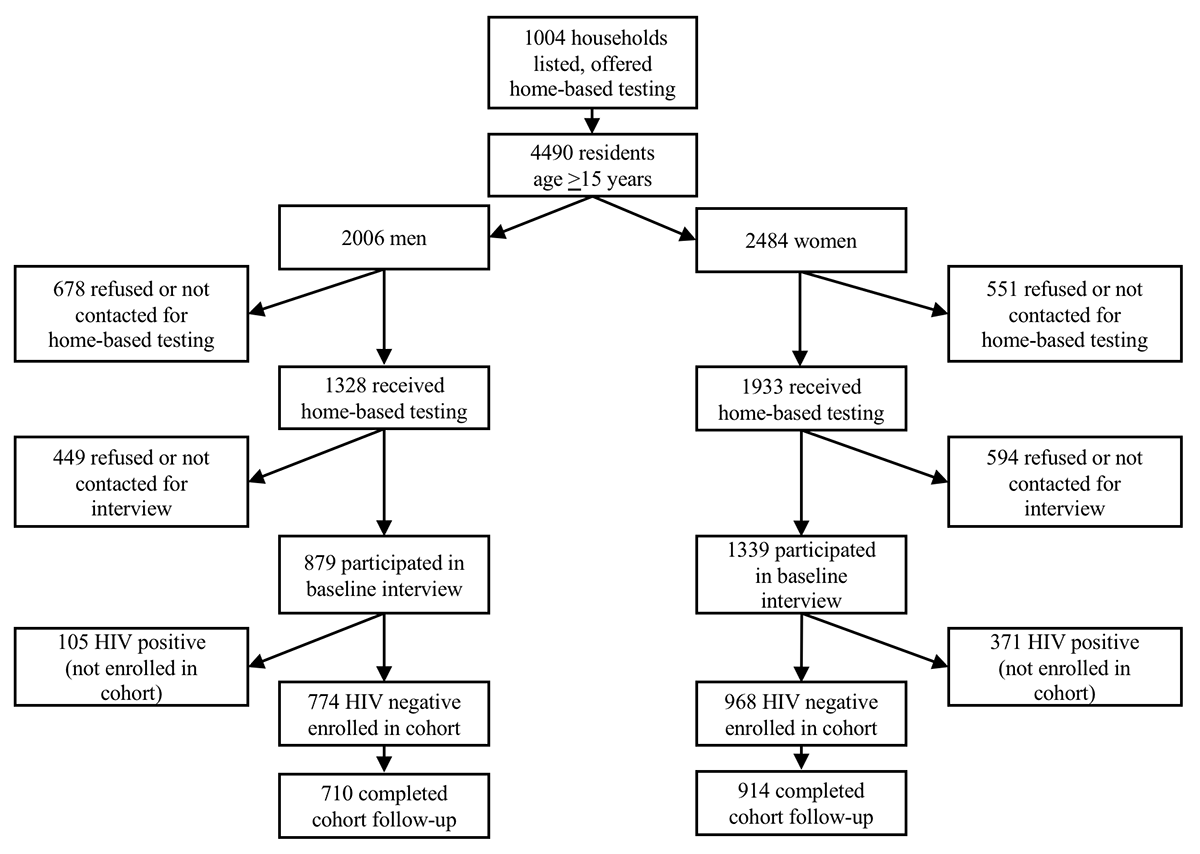
Flow diagram of household listing, receipt of home-based HIV testing, participation in the cohort study and follow-up measurements among adults age ≥ 15 years in five community-based sites of the Zambezi region of Namibia, 2014-2016.

The TCE program offered home-based testing to 1004 households across the 5 sites (Figure 1). Among persons aged ≥15 years residing in these households, 72.63% (3261/4490) received home-based testing, of whom 68.02% (2218/3261) completed the baseline interview. Among HIV-negative persons who participated in the baseline interview, 93.2% (1624/1742) completed the follow-up HIV test and interview. The median follow-up time was 433 days (IQR 397-478), which was notably higher than the intended follow-up time of 365 days. There were no significant differences in follow-up time by age, sex, or urban vs rural sites. Women were more likely than men to receive home-based testing, agree to the baseline interview, and be retained for follow-up.

### Description of Cohort Participants

Demographic characteristics and HIV-related risk behaviors of the cohort participants who completed the follow-up are shown in Table 1. Young women aged 15 to 24 years comprised 43.6% (398/914) of female participants, 25.2% (230/914) lived in the urban Ngweze site, 65.5% (599/914) had not tested for HIV in the 12 months before baseline, and 0.9% (8/914) had engaged in transactional sex in the 12 months before baseline. Key characteristics among men were 5.9% (42/710) being aged 40 to 44 years, 11.1% (79/710) seeking HIV testing outside the study in the year before follow-up (ie, in addition to the testing provided by the study), and 4.6% (32/710) self-reporting circumcision before baseline. Among HIV-negative participants who tested with their partner, 8.6% (40/463) had an HIV-positive partner, of whom 28% (11/40) were on ART. Quality assurance through retesting DBS from baseline and follow-up participants detected no misclassification of serostatus. All counselors scored 100% on the rapid testing proficiency panels during the study.

**Table 1:** Demographic and behavioral characteristics of HIV-negative participants who completed baseline and follow-up measurements—household cohort study of adults aged ≥15 years in the Zambezi region of Namibia, 2014 to 2016 (N=1624).

Variable		Total, n (%)	Women (n=914), n (%)	Men (n=710), n (%)
**Age (years)^a^**				
	15-19	301 (18.53)	176 (19.3)	125 (17.6)
	20-24	379 (23.34)	222 (24.3)	157 (22.1)
	25-29	253 (15.58)	134 (14.7)	119 (16.8)
	30-34	195 (12.01)	107 (11.7)	88 (12.4)
	35-39	152 (9.36)	69 (7.5)	83 (11.7)
	40-44	92 (5.67)	50 (5.5)	42 (5.9)
	45-49	63 (3.88)	30 (3.3)	33 (4.6)
	50-64	189 (11.64)	126 (13.8)	63 (8.9)
**Site^a^**				
	Ngweze urban	384 (23.65)	230 (25.2)	154 (21.7)
	Mavuluma urban	356 (21.92)	217 (23.7)	139 (19.6)
	Bukalo rural	368 (22.66)	175 (19.1)	193 (27.2)
	Ngoma rural	201 (12.38)	101 (11.1)	100 (14.1)
	Sibbinda rural	315 (19.40)	191 (20.9)	124 (17.5)
**Residence^a^**				
	Rural	887 (54.62)	468 (51.2)	419 (59.0)
	Urban	737 (45.38)	446 (48.8)	291 (41.0)
**Age (years) and residence^a^**				
	15-24, rural	328 (20.20)	169 (18.5)	159 (22.4)
	15-24, urban	354 (21.80)	229 (25.1)	125 (17.6)
	≥25, rural	559 (34.42)	299 (32.7)	260 (36.6)
	≥25, urban	383 (23.58)	217 (23.7)	166 (23.4)
Currently married^a^		644 (39.66)	381 (41.7)	263 (37.0)
Tested for HIV in the 12 months before enrollment^a^		485 (29.86)	315 (34.5)	170 (23.9)
Tested with a partner at enrollment^a^		463 (28.51)	264 (28.9)	199 (28.0)
Had a serodiscordant positive partner (among those tested with a partner at enrollment)^a^		40 (8.6)	13 (4.9)	27 (13.6)
Partner on antiretroviral treatment (among those with serodiscordant positive testing partner)^a,^^b^		11 (27.5)	4 (30.8)	7 (25.9)
Circumcised (among men only)^a^		N/A^c^	N/A	32 (4.6)
Sought HIV testing outside the study in past 12 months^d^		212 (13.05)	133 (14.6)	79 (11.1)
Had sex partner residing outside study area in the past 12 months^b,^^d^		144 (11.70)	84 (12.4)	60 (10.8)
Engaged in transactional sex in the past 12 months^d^		44 (2.71)	8 (0.9)	36 (5.1)
Used a condom at the last sexual encounter^b,^^d^		677 (54.82)	381 (56.0)	296 (53.3)
Used condoms consistently with all sex partners in past the 12 months^b,^^d^		119 (9.64)	58 (8.5)	61 (11.0)
Had multiple sex partners in the past 12 months^d^		38 (2.34)	12 (1.3)	26 (3.7)

^a^Data collected at baseline.

^b^Among participants who reported having any sex partners between baseline and follow-up (n=1235, including 680 women and 555 men).

^c^N/A: not applicable.

^d^Data collected at follow-up.

### Rates of HIV Incidence

**Figure 2:**
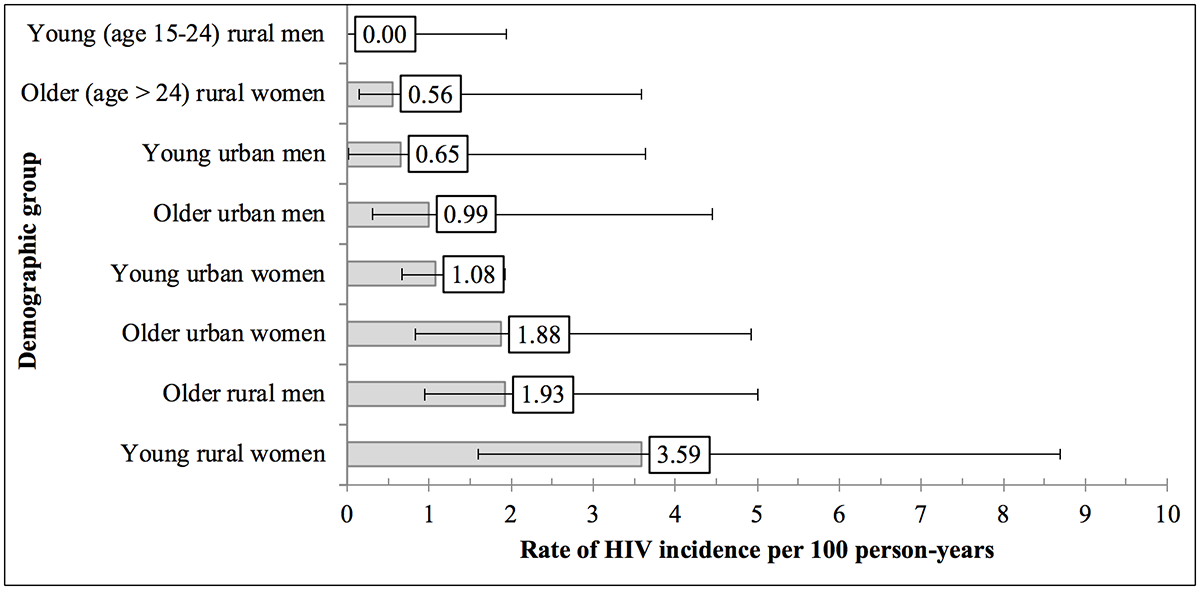
HIV incidence per 100 person-years by age, residence, and sex; household cohort of adults age ≥ 15 years in the Zambezi region of Namibia, 2014-2016 (N=1624). Error bars in the figure represent two-sided 95% CI, except when there were 0 seroconversions, in which case CI are one-sided and 97.5%.

There were 26 seroconversions in 1954 PY among the 1624 baseline HIV-negative participants who completed the follow-up (Table 2), equating to an overall incidence rate of 1.33 per 100 PY (95% CI 0.91-1.95). When pooled across age groups, the overall incidence was not significantly higher for women (1.55 per 100 PY, 95% CI 1.12-2.17; *P*=.26) relative to men (1.05 per 100 PY, 95% CI 0.54-2.31). Among women, most seroconversions occurred in the younger age groups, with 10 out of 17 among women aged 15 to 24 years and 5 among those aged 15 to 19 years (2.42 per 100 PY, 95% CI 0.97-7.34). Among men, the incidence was highest among those aged 40 to 44 years (8.21 per 100 PY, 95% CI 3.76-21.14). When participants were grouped into 8 demographic categories by sex, age (15-24 vs 25 and above), and residence (urban vs rural; Table 2 and Figure 2), the highest incidence was among rural adolescent girls and young women (AGYW) aged 15 to 24 years (3.59 per 100 PY, 95% CI 1.60-8.69). Rural, older men (>25 years) had the second highest incidence (1.93 per 100 PY, 95% CI 0.94-5.01). No seroconversions occurred among men who self-reported being circumcised at baseline (0 per 100 PY, 97.5% CI 0-9.78). No seroconversions occurred among women (0 per 100 PY, 97.5% CI 0-80.02) or men (0 per 100 PY, 97.5% CI 0-45.26) who had an HIV-positive partner on ART.

**Table 2:** HIV incidence per 100 person-years by sex and demographic and behavioral characteristics—household cohort study of adults aged ≥15 years in the Zambezi region of Namibia, 2014 to 2016 (N=1624).

Variable		Women			Men		
		Incident infections	Rate per 100 person, years (CI)^a^	*P* value	Incident infections	Rate per 100 person, years (CI)	*P* value
Overall		17	1.55 (1.12-2.17)	.29	9	1.05 (0.54-2.31)	Ref^b^
**Age (years)^c^**							
	15-19	5	2.42 (0.97-7.34)	.42	0	0.00 (0.00-2.44)	—^d^
	20-24	5	1.88 (1.05-3.57)	.68	1	0.53 (0.01-2.93)	Ref
	25-29	2	1.23 (0.31-8.52)	Ref	1	0.69 (0.02-3.87)	.80
	30-34	4	3.09 (1.68-6.61)	.40	2	1.86 (0.50-12.51)	.25
	35-39	0	0.00 (0.00-4.53)	—	1	1.00 (0.03-5.57)	.60
	40-44	0	0.00 (0.00-6.25)	—	4	8.21 (3.76-21.14)	<.001
	45-49	1	2.89 (0.07-16.10)	.61	0	0.00 (0.00-9.23)	—
	50-64	0	0.00 (0.00-2.34)	—	0	0.00 (0.00-4.95)	—
**Currently married^c^**							
	No	14	2.20 (1.38-3.49)	.03	6	1.12 (0.49-2.87)	.79
	Yes	3	0.65 (0.25-2.21)	Ref	3	0.94 (0.38-3.08)	Ref
**Site^c^**							
	Ngweze urban	5	1.88 (1.23-3.02)	<.001	1	0.58 (0.01-3.21)	Ref
	Mavuluma urban	3	1.17 (0.41-3.86)	Ref	2	1.29 (0.16-4.64)	.75
	Bukalo rural	3	1.47 (0.41-5.40)	.36	3	1.31 (0.27-3.84)	.58
	Ngoma rural	2	1.59 (1.46-1.76)	.005	2	1.61 (1.34-1.96)	.34
	Sibbinda rural	4	1.81 (0.75-5.83)	.11	1	0.70 (0.02-3.89)	.94
**Residence^c^**							
	Rural	9	1.62 (0.99-2.76)	.53	6	1.20 (0.51-3.60)	.54
	Urban	8	1.47 (0.91-2.48)	Ref	3	0.85 (0.23-5.36)	Ref
**Age (years) and residence^c^**							
	15-24 and rural	7	3.59 (1.60-8.69)	.04	0	0.00 (0.00-1.94)	—
	15-24 and urban	3	1.08 (0.66-1.93)	.58	1	0.65 (0.02-3.64)	Ref
	≥25 and rural	2	0.56 (0.14-3.59)	Ref	6	1.93 (0.94-5.01)	.08
	≥25 and urban	5	1.88 (0.83-4.92)	.18	2	0.99 (0.31-4.48)	.36
**Tested for HIV in the 12 months before enrollment^c^**							
	No	14	1.97 (1.32-2.95)	.05	5	0.77 (0.32-2.31)	.11
	Yes	3	0.78 (0.33-2.41)	Ref	4	1.95 (1.03-4.24)	Ref
**Tested with a partner at enrollment^c^**							
	No	16	2.05 (1.41-2.98)	.05	7	1.15 (0.54-2.75)	.57
	Yes	1	0.30 (0.01-1.69)	Ref	2	0.82 (0.22-5.47)	Ref
**Had a serodiscordant positive partner (among those tested with a partner)^c^**							
	No	0	0.00 (0.00-1.18)	—	1	0.47 (0.01-2.63)	Ref
	Yes	1	6.82 (0.17-38.01)	Ref	1	3.06 (0.08-17.03)	.23
**Partner on antiretroviral therapy (among those with serodiscordant positive partner)^c^**							
	No	1	9.94 (0.25-55.38)	Ref	1	4.07 (0.10-22.68)	Ref
	Yes	0	0.00 (0.00-80.02)	—	0	0.00 (0.00-45.26)	—
**Circumcised (among men only)^c^**							
	No	N/A^e^	N/A	—	9	1.13 (0.58-2.49)	Ref
	Yes	N/A	N/A	—	0	0.00 (0.00-9.78)	—
**Sought testing for HIV outside the study in past 12 months^f^**							
	No	11	1.17 (0.68-2.12)	.14	4	0.53 (0.17-2.42)	.03
	Yes	6	3.72 (1.64-9.27)	Ref	5	5.23 (1.99-16.65)	Ref
**Had a sex partner residing outside of study area in past 12 months^f^**							
	No	13	1.82 (1.31-2.57)	.13	4	0.67 (0.30-1.73)	.05
	Yes	3	2.98 (1.62-6.71)	Ref	2	2.79 (0.34-10.07)	Ref
**Engaged in transactional sex in the past 12 months^f^**							
	No	15	1.38 (0.88-2.24)	.01	9	1.11 (0.55-2.51)	Ref
	Yes	2	22.75 (3.79-100)	Ref	0	0.00 (0.00-8.52)	—
**Used a condom at the last sexual encounter^c^**							
	No	9	2.52 (1.15-5.08)	.37	1	0.32 (0.08-1.76)	.23
	Yes	7	1.53 (0.84-3.03)	Ref	5	1.41 (0.45-7.42)	Ref
**Used condoms consistently with all sex partners in the past 12 months^f^**							
	No	14	1.88 (1.45-2.48)	.98	7	1.21 (0.62-2.72)	.64
	Yes	2	2.82 (0.67-20.52)	Ref	1	0.87 (0.02-4.60)	Ref
**Had multiple sex partners in the past 12 months^f^**							
	No	15	1.38 (0.89-2.25)	.02	9	1.09 (0.56-2.41)	Ref
	Yes	2	14.78 (3.81-94.41)	Ref	0	0.00 (0.00-12.07)	—

^a^CIs are cluster-robust unless there are 1 or 0 seroconversions, in which case the CI is Poisson exact. CI is 2-sided 95% except when there are 0 seroconversions, in which cases CI is 1-sided 97.5%.

^b^*Ref* is the reference group for Cox models.

^c^Data collected at baseline.

^d^*P* values were not calculated when there were 0 seroconversions.

^e^N/A: not applicable.

^f^Data collected at follow-up.

### Correlates of HIV Incidence

In the multivariable model for women (Table 3), factors associated with increased risk for seroconversion were AGYW residing in rural sites vs other women (aHR 4.26, 95% CI 1.39-13.13; *P*=.01), residing in the Ngweze urban site vs other sites (aHR 2.34, 95% CI 1.25-4.40; *P*=.01), not testing for HIV in the 12 months preceding baseline vs testing (aHR 3.38, 95% CI 1.04-10.95, *P*=.05), and engaging in transactional sex vs no transactional sex (aHR 17.64, 95% CI 2.88-108.14; *P*=.02). In the multivariable model for men, the risk of seroconversion was higher among those aged 40 to 44 years relative to other age groups (aHR 13.04, 95% CI 5.98-28.41; *P*<.001). Men who sought HIV testing between baseline and follow-up outside of the study also had a higher risk for seroconversion than men who had not sought testing between baseline and follow-up (aHR 8.28, 95% CI 1.39-49.38; *P*=.02). No multicollinearity among the variables in the models was observed.

**Table 3:** Correlates of HIV seroconversion among women and men, multivariable Cox proportional hazards models, and household cohort study of adults aged ≥15 years in the Zambezi region of Namibia, 2014 to 2016 (N=1624).

Variable		Full model, adjusted hazards ratio (95% CI)^a^	*P* value	Final model, adjusted hazards ratio (95% CI)^a^	*P* value
**Women**					
	15-24 years old and resident of rural site (vs other age and residential groups)^b^	4.17 (1.37-12.65)	.01	4.26 (1.39- 13.13)	.01
	Resident of Ngoma rural (vs residents of other sites)^b^	0.55 (0.05-5.70)	.62	—^c^	—
	Resident of Ngweze urban (vs residents of other sites)^b^	2.13 (1.08-4.18)	.03	2.34 (1.25- 4.40)	.01
	Not currently married (vs married)^b^	1.34 (0.58-3.07)	.49	—	—
	Not tested with partner at enrollment (vs tested with partner)^b^	5.95 (0.65-54.3)	.13	—	—
	Not tested for HIV in the 12 months before enrollment (vs tested)^b^	3.12 (0.91-10.68)	.07	3.38 (1.04-10.95)	.05
	Engaged in transactional sex (vs did not engage in transactional sex)^d^	10.33 (2.48- 42.95)	.001	17.64 (2.88-108.14)	.02
	Had multiple sex partners in the past 12 months (vs did not have multiple partners)^d^	3.17 (0.58-17.48)	.19	—	—
**Men**					
	Age 40-44 years (vs other age groups)^b^	6.90 (2.75-17.34)	<.001	13.04 (5.98-28.41)	<.001
	Older and residing in a rural site (vs other age and residential groups)^b^	7.90 (0.65-96.49)	.11	—	—
	Sought testing for HIV outside the study in the past 12 months (vs did not seek testing)^d^	35.23 (12.40-100.06)	<.001	8.28 (1.39-49.38)	.02
	Had a sex partner residing outside the study area (vs did not have partner outside study area)^d^	2.31 (0.68-7.88)	.18	—	—

^a^All CIs are 2-sided 95% and cluster robust.

^b^ Data collected at baseline.

^c^Variables at *P*≤.10 in the bivariate models (Table 2) were included in the multivariable Cox models. Variables at *P*>.10 in the full model were removed for the final model. Variables at *P*<.05 in the final multivariable models were considered statistically significant.

^d^Data collected at follow-up.

## Discussion

### Principal Findings

Our longitudinal, sentinel cohort study reports the first directly observed measure of HIV incidence in the adult population of Zambezi, Namibia. Nearly three decades into Namibia’s epidemic, this is the first estimate of incidence for its most severely affected region. Our measure of 1.33 per 100 PY, compared with modeled HIV incidence for all Namibia during this period (0.78 per annum) [1], corroborates that Zambezi is a region where higher levels of HIV transmission persist. Our method, which uses an existing community-based testing program, is a replicable framework for sentinel HIV incidence surveillance that can be used in the absence of or supplemental to data obtained from other methods.

We were able to detect significant correlates of HIV seroconversion that can be used to understand the extent to which existing HIV prevention interventions are working and where additional interventions should be delivered. These include where to prioritize the deployment and scale-up of effective biomedical interventions such as enhanced test and treatment strategies and PrEP. AGYW living in rural areas had more than four times the likelihood of acquiring HIV infection compared with other women. HIV incidence among men was highest in the 40- to 44-year-old group and among older men in rural areas. These findings are consistent with a pattern of intergenerational, heterosexual transmission observed across sub-Saharan Africa [1,8,9,25], which may be explained by the early sexual debut in AGYW, harmful gender norms, transactional sex, and income disparities in sexual relationships [25,27]. The latter two hypotheses are supported by our study’s observation that transactional sex was a significant predictor of seroconversion, and by the extremely high prevalence observed among female sex workers in a separate cross-sectional study in the Zambezi region [28]. We also observed that men who sought HIV testing outside of the study between baseline and follow-up were more likely to seroconvert, suggesting men who seek frequent testing may be correctly perceiving themselves to be at elevated risk. The finding stood in contrast to women; those who did not have a history of a test before baseline were more likely to seroconvert. Women may be less likely to perceive their risk of infection (eg, their risk is from their husbands’ or regular partners’ behaviors), highlighting the need for home-based, provider-initiated, or other forms of testing to reach women who do not seek testing on their own. High HIV incidence was also observed in the Ngweze urban site. Multiple cases within this small neighborhood may suggest that we found a hotspot of transmission, highlighting the potential yield of index client partner tracing for case detection. Alternatively, the high incidence in this neighborhood may be correlated with another factor, in which case area mobile testing may diagnose additional cases. Although we observed no seroconversions among circumcised men and persons whose partners were on ART, the sample sizes were small, and we were unable to test for significance in our models. Future applications of this surveillance method would need to enroll a larger sample to assess whether the population-level prevention effects of these biomedical interventions are consistent with those observed in randomized controlled trials [6,7,29]. In summary, our results point to specific sexual risk and health-seeking behaviors that can be prioritized for enhanced behavioral and biomedical prevention interventions, particularly focusing on the populations and areas in the Zambezi region identified as having a higher incidence.

### Comparison With Prior Work

Few recent direct measures of HIV incidence are available from longitudinal studies elsewhere in sub-Saharan Africa. HIV incidence was 2.4 per 100 PY (95% CI 2.00-2.54) in a national population-based cohort in Eswatini from 2010 to 2011 [8], 0.27 per 100 PY (95% CI 0.18-0.35) in a national population-based cohort in Rwanda from 2013 to 2014 [9], 1.11 per 100 PY (95% CI 0.91-1.31) in a regional population-based cohort in Gem, western Kenya from 2006 to 2016 [10], and 0.55 per 100 PY (95% CI 0.45-0.66) in a study in rural Uganda that measured HIV incidence through home-based testing campaigns across two rounds in 2006 and 2008 [22]. The only other longitudinal measure of HIV incidence from Namibia was 2.4 per 100 PY (95% CI 1.9-2.9) in a household-based study in Windhoek from 2007 to 2009 [23], a time when few PLHIV were on ART [1,3]. In an era of working to achieve HIV epidemic control worldwide, more incidence estimates from cohorts such as these are needed to assess prevention efforts and target hotspots of continuing transmission. A longitudinal sentinel incidence surveillance approach similar to ours can strike a balance of efficiency and rigor by leveraging existing HIV testing programs in high-risk areas and populations below the national level.

### Strengths and Limitations

Our longitudinal sentinel incidence surveillance study points to moderately high internal validity (eg, the robustness of correlates of HIV acquisition within the sentinel population). Nearly three-fourths (3261/4490, 72.63%) of residents accepted home-based testing by TCE, of whom 68.02% (2218/3261) participated in our cohort. Participation was lower than that observed in the Eswatini (73.8%) [8], western Kenya (82.6%) [10], Rwanda (98.4%) [9], and Windhoek cohorts (88%) [23]. Nonetheless, our retention rate of 93.23% (1624/1742) was comparable with or higher than 41.3% in western Kenya [10], 58.0% in Windhoek [23], 64.4% in rural Uganda [22], 91.7% in Rwanda [9], and 94.4% in Eswatini [8]. Moderate levels of participation and high levels of retention in our cohort led to an overall incidence estimate that was reasonably precise (95% CI 0.91-1.95). However, greater precision and power to detect differences in incidence between subgroups may have been possible if more residents had participated in our cohort.

Although our results are encouraging that sentinel surveillance integrated within existing testing programs can track HIV incidence and demonstrate prevention impact, we recognize limitations. First, the sample size and few incident infections resulted in low precision for HIV incidence in subgroups, low statistical power to detect smaller effects for HIV acquisition, and an increased chance that some correlates may be because of chance. As the incidence is declining in the current era, larger sample sizes are needed to measure the impact of prevention programs. Nonetheless, the sentinel incidence surveillance approach has two advantages for increasing statistical power: purposely choosing populations with high HIV incidence and leveraging programs already testing large numbers of persons at risk. If community-based testing programs are already in place, the sentinel approach can be scaled up to include more sites with minimal additional resources, forming an integrated national system similar to antenatal clinic sentinel surveillance for HIV prevalence [30]. A second limitation is representativeness, affected by the choice of sites and by lower participation for some groups, including men, who are consistently less likely to be tested for HIV than women in settings across Africa [31]. Our incidence estimates and factor analyses among men may be biased if those who participated had different risk profiles than those who did not. Furthermore, men in our study were not asked if they had sex with other men. As such, we were not able to assess behavior among men who have sex with men (MSM) as a potential correlation of seroconversion. Other studies have shown a high prevalence among MSM in Windhoek, but the prevalence is approximately equal among MSM and the general population in less densely populated areas outside the capital [32]. Given that our study setting more closely resembles those less densely populated areas, we believe the potential biases of noninclusion or nonself-identification of MSM in our cohort to be likely low.

By design, we deliberately chose the sentinel population within the most severely affected region of Namibia and purposively selected a limited number of clusters for the sake of efficiency. Unlike the studies in Eswatini [8] and Rwanda [9], our estimates do not extrapolate to the national level. Unfortunately, data on the characteristics of clients reached by the TCE program in nonsampled areas of Zambezi were not available for analysis. Although we assume that the demographic and risk profiles of residents in sampled and nonsampled urban and rural areas across the region are comparable, we were not able to confirm this assumption and its effect on the generalizability of our results. Nonetheless, our sentinel approach produced a precise estimate for a high-priority subnational area. Moreover, the design and intention of the sentinel surveillance approach are to select sites that can provide early signals of changes in the epidemic over person, place, and time. A third limitation is that we depended upon having a large-scale, pre-existing community-based HIV testing program. The TCE program was funded to test the entire Zambezi population using a door-to-door home-based approach, presenting an opportunity to coordinate longitudinal sentinel incidence surveillance across a defined geographic area with minimal additional resources. The sentinel incidence surveillance approach may require the identification of other programs that conduct repeat HIV testing in defined populations or within consistent catchment areas. Fourth, participation rates in the TCE program and cohort leave room for potential bias with reduced external validity. Finally, the act of counseling and testing for HIV at baseline and the anticipation of follow-up testing may reduce risk behavior and therefore underestimate HIV incidence relative to the surrounding population.

### Conclusions

We tested an efficient method to obtain a directly observed, longitudinal measure of HIV incidence in a high-prevalence region of Namibia. Nearly three decades into Namibia’s epidemic, this is the first estimate of the incidence for this region. With the achievement of its target sample, high retention, and ability to detect correlates of seroconversion, our approach appears to be a viable community-based surveillance method that could be replicated in other settings serviced by similar testing programs. We believe this approach can strike a reasonable balance between the additional resources required and the ability to generate direct measures of prevention impact. As HIV testing becomes increasingly accessible and frequent, more opportunities to measure incidence through active and passive repeat testing will arise. Longitudinal sentinel incidence surveillance can be integrated into other community-based programs or facilities conducting high numbers of repeat HIV tests, such as antenatal and sexually transmitted infection clinics [16,33,34], and those servicing key populations at high risk for HIV. The hard-won tools to treat and prevent HIV have placed epidemic control and elimination within reach. We need to take every opportunity to demonstrate and ensure that they are working.

## Abbreviations

AGYW: adolescent girls and young women
aHR: adjusted hazard ratio
ART: antiretroviral treatment
CDC: Centers for Disease Control and Prevention
DBS: dried blood spot
MSM: men who have sex with men
PLHIV: people living with HIV
PrEP: pre-exposure prophylaxis
PY: person-years
TCE: Total Control of the Epidemic

## References

[1] (2018). Joint United Nations Programme on HIV/AIDS.

[2] The Nambia Ministry of Health and Social Services (2014). The DHS Program.

[3] (2008). AIDSinfo: UNAIDS.

[4] UNAIDS (2016). World Health Organization.

[5] The United States President's Emergency Plan for AIDS Relief (2017). US Embassy in Namibia.

[6] Gray RH, Kigozi G, Serwadda D, Makumbi F, Watya S, Nalugoda F, Kiwanuka N, Moulton LH, Chaudhary MA, Chen MZ, Sewankambo NK, Wabwire-Mangen F, Bacon MC, Williams CF, Opendi P, Reynolds SJ, Laeyendecker O, Quinn TC, Wawer MJ (2007). Male circumcision for HIV prevention in men in Rakai, Uganda: a randomised trial. Lancet.

[7] Weidner W (2007). Words of wisdom re: male circumcision for HIV prevention in young men in Kisumu, Kenya: a randomised controlled trial. Eur Urol.

[8] Justman J, Reed JB, Bicego G, Donnell D, Li K, Bock N, Koler A, Philip NM, Mlambo CK, Parekh BS, Duong YT, Ellenberger DL, El-Sadr WM, Nkambule R (2017). Swaziland HIV incidence measurement survey (SHIMS): a prospective national cohort study. Lancet HIV.

[9] Nsanzimana S, Remera E, Kanters S, Mulindabigwi A, Suthar AB, Uwizihiwe JP, Mwumvaneza M, Mills EJ, Bucher HC (2017). Household survey of HIV incidence in Rwanda: a national observational cohort study. Lancet HIV.

[10] Borgdorff MW, Kwaro D, Obor D, Otieno G, Kamire V, Odongo F, Owuor P, Muthusi J, Mills LA, Joseph R, Schmitz ME, Young PW, Zielinski-Gutierrez E, de Cock KM (2018). HIV incidence in western Kenya during scale-up of antiretroviral therapy and voluntary medical male circumcision: a population-based cohort analysis. Lancet HIV.

[11] Williams B, Gouws E, Wilkinson D, Karim SA (2001). Estimating HIV incidence rates from age prevalence data in epidemic situations. Stat Med.

[12] Downs AM, Heisterkamp SH, Ravà L, Houweling H, Jager JC, Hamers FF (2000). Back-calculation by birth cohort, incorporating age- specific disease progression, pre-AIDS mortality and change in European AIDS case definition. European union concerted action on multinational AIDS scenarios. AIDS.

[13] Hallett TB, Zaba B, Todd J, Lopman B, Mwita W, Biraro S, Gregson S, Boerma JT, ALPHA Network (2008). Estimating incidence from prevalence in generalised HIV epidemics: methods and validation. PLoS Med.

[14] Guy R, Gold J, Calleja JM, Kim AA, Parekh B, Busch M, Rehle T, Hargrove J, Remis RS, Kaldor JM (2009). Accuracy of serological assays for detection of recent infection with HIV and estimation of population incidence: a systematic review. Lancet Infect Dis.

[15] Pappaioanou M, Dondero TJ, Petersen LR, Onorato IM, Sanchez CD, Curran JW (1990). The family of HIV seroprevalence surveys: objectives, methods, and uses of sentinel surveillance for HIV in the United States. Public Health Rep.

[16] World Health Organization (2003). Guidelines for Conducting HIV Sentinel Serosurveys among Pregnant Women and Other Groups.

[17] WHO, PEPFAR, CDC, USAID, LSTM (2012). Planning, Implementing and Monitoring Home-Based HIV Testing and Counselling: A Practical Handbook for Sub-Saharan Africa.

[18] World Health Organization (2012). Service Delivery Approaches to HIV Testing and Counselling (HTC): A Strategic Policy Framework.

[19] Bateganya M, Abdulwadud O, Kiene S (2010). Home-based HIV voluntary counselling and testing (VCT) for improving uptake of HIV testing. Cochrane Database Syst Rev.

[20] Tabana H, Nkonki L, Hongoro C, Doherty T, Ekström AM, Naik R, Zembe-Mkabile W, Jackson D, Thorson A (2015). A cost-effectiveness analysis of a home-based HIV counselling and testing intervention versus the standard (facility based) HIV testing strategy in rural South Africa. PLoS One.

[21] Naik R, Tabana H, Doherty T, Zembe W, Jackson D (2012). Client characteristics and acceptability of a home-based HIV counselling and testing intervention in rural South Africa. BMC Public Health.

[22] Okiria AG, Okui O, Dutki M, Baryamutuma R, Nuwagaba CK, Kansiime E, Ojamuge G, Mugweri J, Fleuret J, King R, Bazeyo W, Lindan C (2014). HIV incidence and factors associated with seroconversion in a rural community home based counseling and testing program in eastern Uganda. AIDS Behav.

[23] Aulagnier M, Janssens W, de Beer I, van Rooy G, Gaeb E, Hesp C, van der Gaag J, de Wit TF (2011). Incidence of HIV in Windhoek, Namibia: demographic and socio-economic associations. PLoS One.

[24] Rogers WH (1994). Regression standard errors in clustered samples. Stata Tech Bull.

[25] Harrison A, Colvin CJ, Kuo C, Swartz A, Lurie M (2015). Sustained high HIV incidence in young women in southern Africa: social, behavioral, and structural factors and emerging intervention approaches. Curr HIV/AIDS Rep.

[26] Chen X, Ender P, Mitchell M, Wells C (2003). IDRE Stats-Statistical Consulting Web Resources.

[27] Dellar RC, Dlamini S, Karim QA (2015). Adolescent girls and young women: key populations for HIV epidemic control. J Int AIDS Soc.

[28] (2015). UC Global Programs.

[29] Cohen MS, Chen YQ, McCauley M, Gamble T, Hosseinipour MC, Kumarasamy N, Hakim JG, Kumwenda J, Grinsztejn B, Pilotto JH, Godbole SV, Mehendale S, Chariyalertsak S, Santos BR, Mayer KH, Hoffman IF, Eshleman SH, Piwowar-Manning E, Wang L, Makhema J, Mills LA, de Bruyn G, Sanne I, Eron J, Gallant J, Havlir D, Swindells S, Ribaudo H, Elharrar V, Burns D, Taha TE, Nielsen-Saines K, Celentano D, Essex M, Fleming TR, HPTN 052 Study Team (2011). Prevention of HIV-1 infection with early antiretroviral therapy. N Engl J Med.

[30] (2016). Ministry of Health and Social Services.

[31] Justman JE, Mugurungi O, El-Sadr WM (2018). HIV population surveys-bringing precision to the global response. N Engl J Med.

[32] (2014). UC Global Programs.

[33] Weinstock H, Sweeney S, Satten GA, Gwinn M (1998). HIV seroincidence and risk factors among patients repeatedly tested for HIV attending sexually transmitted disease clinics in the United States, 1991 to 1996. STD Clinic HIV seroincidence study group. J Acquir Immune Defic Syndr Hum Retrovirol.

[34] Fernyak SE, Page-Shafer K, Kellogg TA, McFarland W, Katz MH (2002). Risk behaviors and HIV incidence among repeat testers at publicly funded HIV testing sites in San Francisco. J Acquir Immune Defic Syndr.

